# Relation between Baseline Total Serum Cortisol Level and Outcome in Pediatric Intensive Care Unit

**DOI:** 10.1038/s41598-019-42443-z

**Published:** 2019-04-12

**Authors:** Osama E. Bekhit, Shereen A. Mohamed, Remon M. Yousef, Hoiyda A. AbdelRasol, Nirvana A. Khalaf, Fatma Salah

**Affiliations:** 1 Pediatric department, Faculty of Medicine, Fayoum University, Fayoum, Egypt; 2 Pediatric department, Kasr AlAiny Faculty of Medicine, Cairo University, Cairo, Egypt; 3 Clinical Pathology department, Faculty of Medicine, Fayoum University, Fayoum, Egypt; 4 Clinical pathology unit, Research institute of ophthalmology, Giza, Egypt

## Abstract

Elevated cortisol level is an component of the stress response. However, some patients have low cortisol levels; a condition termed: critical illness-related corticosteroid insufficiency (CIRCI). Basal cortisol levels during PICU admission may be related to outcome. This prospective cohort study aimed to assess basal total serum cortisol levels and their relation to outcome in PICU. The study included 81 children over 6 months. Total serum cortisol was assessed using an early morning sample. The severity of illness was assessed using the PRISM-III score. Outcome measures included mechanical ventilation duration, use of inotropic support, length of stay, mortality. Comparison between patients’ subgroups according to total serum cortisol levels revealed significantly higher PRISM-III score in patients with total serum cortisol levels. In addition, those patients had a significantly higher mortality rate when compared with patients with low and normal total serum cortisol levels. Multivariate logistic regression analysis recognized high total serum cortisol level and PRISM-III score as significant predictors of mortality. We concluded that PRISM-III score and elevated total serum cortisol levels are significant predictors of mortality in the PICU. Although CIRCI is prevalent in this population, it wasn’t associated with an increased mortality rate.

## Introduction

Critical illness entails a complex pathological process that triggers exaggerated inflammatory and stress responses together with systemic dysregulation of endocrine functions. These factors –if not properly managed- ultimately result in impaired tissue perfusion and multi-organ failure^1^. In the context of endocrine response to critical illness, increased levels of the stress hormone cortisol serve to control the stress response and maintain cardiovascular homeostasis^2^.

However, in some situations, cortisol levels fail to surge in response to critical diseases. In 2008, an international task force adopted the term: critical illness-related corticosteroid insufficiency (CIRCI) to describe this condition. Probable causes include central inhibition of adrenocorticotropic hormone (ACTH) synthesis and altered synthesis or metabolism of cortisol^3^. Diagnosis of CIRCI requires random plasma cortisol level of less than 10 µg/dl or cortisol level rise of less than 9 µg/dl over 60 minutes after ACTH injection^4^.

The relation between cortisol response and ICU outcome remains controversial^5^ with some reports documenting the detrimental effects of very high cortisol levels and others blaming low cortisol levels for unfavorable outcome^6–8^.

In pediatric populations, altered cortisol response constitutes a special challenge to the managing teams. However, this issue was rarely investigated in comparison to the frequently published studies on adult patients^9^.

## Aim of Work

The aim of this study is to assess basal cortisol levels and their relation to outcome of critically ill children.

## Patients and Methods

This prospective cohort study was conducted at pediatric ICUs, Fayoum University Hospital and Aboul-Elrish Pediatric University Hospital, over 6 months period. The study protocol was approved by the Research Ethical Committee of Kasr Alainy Faculty of Medicine, Cairo University and Faculty of Medicine, Fayoum University and in accordance with Declaration of Helsinki for medical research involving human subjects. The research objectives were explained to the patients’ guardians and informed consent was taken before patients’ enrollment from patients’ legal guardians. All patients admitted to PICU, aged from one month to 14 years were included. Patients with known hypothalamic, pituitary, adrenal or severe hepatic diseases, or on corticosteroid treatment or other medications affecting adrenal function in the preceding 3 months were excluded.

Demographic and clinical data were collected. Routine laboratory investigations were performed within the first 24 hours after admission. These included complete blood count, renal profile, coagulation profile, serum albumin, arterial blood gases. Early morning, fasting sample for total serum cortisol was withdrawn. Total serum cortisol levels were assessed using a commercial solid-phase chemiluminescent immunoassay. The reported levels were classified into three categories: <10 µg/dl (CIRCI), 10–34 µg/dl (normal range), and ≥35 µg/dl (above normal range)^10^.

Severity of critical illness was evaluated using pediatric risk of mortality III score (PRISM-III)^11^. Outcome measures included mechanical ventilation duration, use of inotropic support, PICU length of stay, and PICU mortality.

Statistical analysis was performed using SPSS 15.0 (IBM, Chicago, IL, USA). Data are presented as frequency and percent, mean ± SD or median and interquartile range (IQR). Comparative statistics were performed using Mann–Whitney U-test, Kruskal–Wallis test, chi-square test, or Fisher’s exact test as appropriate. Pearson’s or Spearman’s correlation coefficients were used to assess the relationships among variables. Logistic regression analysis was used to detect predictors of mortality. Receiver operator characteristic (ROC) curve analysis was utilized to discover reliability of independent variables to predict outcome. P < 0.05 was considered significant.

## Results

The present study included 81 children admitted to the PICU over a 6 months period. Basic patients’ characteristics are illustrated in Table 1. Comparison between patients’ subgroups classified according to basal total serum cortisol levels revealed significantly higher PRISM-III score in patients with cortisol levels ≥35 µg/dl. In addition, those patients had significantly higher mortality rate when compared with patients with low and normal total serum cortisol levels (39.3% vs 6.7% and 18.4% respectively; p = 0.033). No statistically significant differences were noted between patients’ subgroups regarding other clinical and outcome parameters (Table 2).

**Table 1:** Patients characteristics.

**Age** (months) median (IQR)	9.0 (3.6–30.0)
**Sex** n (%)	
Male	41 (50.6)
Female	40 (49.4)
**Cause of admission** n %	
CNS	10 (12.3)
Respiratory	29 (35.8)
CVS	9 (11.1)
Sepsis	15 (18.5)
Others	18 (22.2)
**PRISM-III** median (IQR)	5.0 (3.0–8.0)
**Total cortisol**	
Median (IRQ)	29.3 (15.2–42.0)
<10 µg/dl n (%)	15 (18.5)
10–34 µg/dl n (%)	38 (46.9)
≥35 µg/dl n (%)	28 (34.6)
**Outcome**	
Hospital stay (days) Median (IQR)	6 (4.0–8.5)
PICU stay (days) Median (IQR)	4 (3–7)
MV (days) Median (IQR)	4 (2.25–4)
MV days % from PICU stay Median (IQR)	20 (0–60)
Inotropic use n (%)	79 (97.5)
Mortality n (%)	19 (23.5)

IQR: interquartile range, CNS: central nervous system, CVS: Cardiovascular system, PICU: Pediatric Intensive Care Unit, MV: Mechanical ventilation. PRISM: Pediatric Risk of Mortality.

**Table 2:** Comparison between patients’ subgroups according to cortisol levels regarding clinical and outcome parameters.

	<10 µg/dl (n = 15)	10–34 µg/dl (n = 38)	≥35 µg/dl (n = 28)	P value
**Age (months)** Median (IQR)	8.0 (3.0–30.0)	13 (4.0–69.0)	7.0 (3.0–13.5)	0.156^#^
**Sex** n (%)				
Male	7 (46.73)	22 (57.9)	12 (42.9)	0.455^##^
Female	8 (53.3)	16 (42.1)	16 (57.1)	
**Cause of admission** n %				
CNS	2 (13.3)	5 (13.2)	3 (10.7)	0.949^##^
Respiratory	8 (53.3)	10 (26.3)	11 (39.3)	0.162^##^
CVS	1 (6.7)	4 (10.5)	4 (14.3)	0.741^##^
Sepsis	2 (13.3)	7 (18.4)	6 (21.4)	0.809^##^
Others	2 (13.3)	12 (31.6)	4 (14.3)	0.163^##^
**PRISM-III** Median (IQR)	4 (0–5.0)	5 (3.0–8.0)	6 (3.0–13.0)	**0.023** ^#*^
**Outcomes**				
Hospital stay (days) Median (IQR)	5 (4–8)	6 (4–7.75)	6 (5–9.75)	0.628^#^
PICU stay (days) Median (IQR)	4 (3–7)	4 (3–6.25)	5 (3–8.75)	0.302
MV (days) Median (IQR)	4 (4–4)	4 (3–4)	3 (2–4)	0.079^#^
MV days % from PICU stay Median (IQR)	0 (0–60)	10 (0–33.93)	55.56 (0–80)	0.277
Inotropic use n (%)	14 (93.3)	38 (100.0)	27 (96.4)	0.333^##^
Mortality n (%)	1 (6.7)	7 (18.4)	11 (39.3)	**0.033** ^##*^

^#^Kruskal Wallis test. ^##^Chi square (χ^2^) test. *Significant.

**Figure 1:**
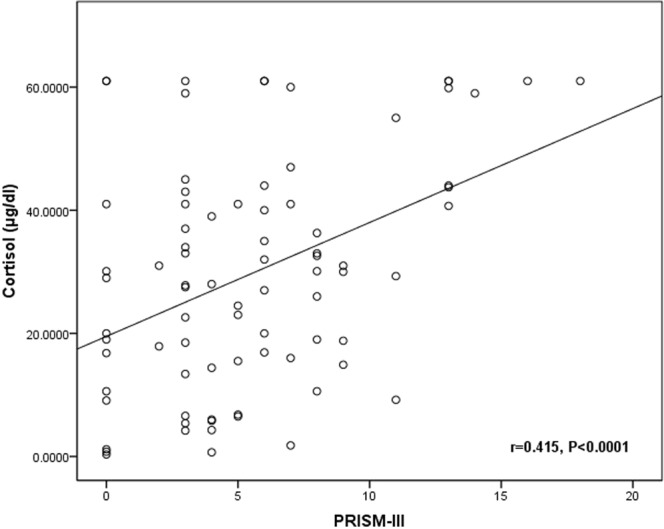
Correlation between Cortisol & PRISM-III.

The relation between mortality and total serum cortisol levels was also confirmed in Table 3 that compared the clinical and outcome variables between survivors and non-survivors. The same table also showed significantly higher PRISM III score in non-survivors when compared with survivors. Moreover, Fig. 1 revealed the significant direct correlation between total serum cortisol levels and PRISM-III score (r = 0.415, p < 0.0001).

**Table 3:** Comparison between survivors and non-survivors regarding clinical and outcome parameters.

	Survivors (n = 62)	Non-survivors (n = 19)	P-value
**Age (months)** Median (IQR)	8.5 (4.0–31.5)	10.0 (3.0–20.0)	0.725^#^
**Sex** n (%)			
Male	33 (53.2)	8 (42.1)	0.396^##^
Female	29 (46.8)	11 (57.9)	
**Cause of admission** n %			
CNS	6 (9.7)	4 (21.1)	0.352^##^
Respiratory	23 (37.5)	6 (31.6)	0.879^##^
CVS	7 (11.3)	2 (10.5)	1.000^##^
Sepsis	11 (17.7)	4 (21.1)	0.979^##^
Others	15 (24.2)	3 (15.8)	0.669^##^
**Cortisol** Median (IQR)	22.8 (10.6–37.5)	43.73 (32.0–59.85)	<**0.0001**^#*^
**PRISM-III** Median (IQR)	4.0 (3.0–6.0)	8.0 (6.0–13.0)	<**0.0001**^#*^
**Outcomes**			
Hospital stay (days) Median (IQR)	6 (4.0–7.0)	7 (3.0–12.0)	0.328^#^
PICU stay (days) Median (IQR)	4 (3–6)	7 (3–11)	0.065
MV days % from PICU stay Median (IQR)	0 (0–46.88)	46.67 (0.0–81.36)	**0.044** ^#*^

^#^Mann-Whitney U test. ^##^Chi square (χ^2^) test. *Significant.

**Figure 2:**
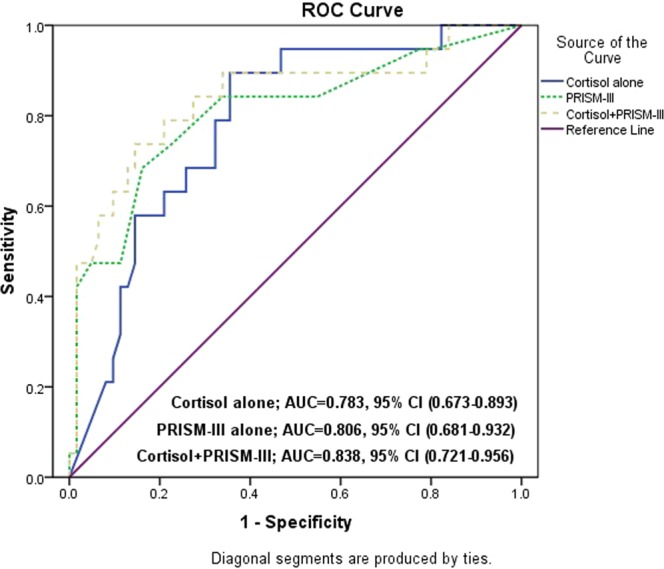
ROC curve for predictors of mortality.

Multivariate logistic regression analysis recognized high basal total serum cortisol level and PRISM-III score as significant predictors of mortality (Table 4). At a cut-off of 30.05, total serum cortisol had a sensitivity, specificity, PPV and NPV of 89.5%, 64.5%, 43.6% and 95.2% respectively for prediction of mortality (AUC: 0.783, 95% CI: 0.673–0.893) while PRISM III score had correspondent values of 84.2%, 66.1%, 43.3% and 93.2 at a cut-off of 5.5 (AUC: 0.806, 95.0% CI: 0.681–0.932). Combined total serum cortisol and PRISM III had an AUC of 0.838 (95% CI: 0.721–0.956) (Table 5, Fig. 2).

**Table 4:** Multiple logistic regression.

	Variables	B	P-value	Odds ratio (OR)	95% CI	
					Lower	Upper
Mortality	Cortisol	0.042	**0.039**	**1.043**	1.002	1.085
	Age	0.002	0.837	1.003	0.987	1.017
	Sex	−0.064	0.922	0.938	0.259	3.399
	PRISM-III	0.258	**0.004**	**1.294**	1.086	1.543
	Constant	−4.313	<0.0001	0.013

**Table 5:** Validity of cortisol and PRISM-III for prediction of mortality.

	Cut-off point	Sensitivity	Specificity	PPV	NPV
Cortisol	30.05	89.5	64.5	43.6	95.2
PRISM-III	5.5	84.2	66.1	43.3	93.2

## Discussion

The reported mortality rate in the present cohort was 23.5%. Previous studies documented mortality rates ranging from 0.5% to 50.0% depending on the underlying disease, its severity, and most importantly the national income and development^12–17^. Our study identified elevated PRISM-III score and elevated total serum cortisol levels as significant predictors of mortality using univariate and multivariate analyses. Moreover, ROC curve analysis revealed that total serum cortisol level of 30 mcg/dL at admission had a good discriminative power for mortality (sensitivity 89.5% and specificity 64.5%).

This is in line with previous conclusions highlighting the relation between PRISM-III score and mortality rate^18,19^. Also, a recent study noted increased mortality rate among children with basal cortisol above 600 nmol/L^20^. Moreover, the study of Nichols *et al*.^21^ on critically ill children with catecholamine dependent septic shock documented an association between higher total serum cortisol levels (≥18 μg/dL) and more severe illness. They also noted that administration of stress dose hydrocortisone in children with low cortisol levels was associated with higher mortality rate. In contrast, another study revealed no correlation between serum cortisol and mortality^9^.

The association between increased cortisol levels and high mortality rate may be explained by the altered cortisol metabolism and disturbed negative feedback mechanism controlling cortisol levels^22^. Probably, these effects are mediated through the rise proinflammatory cytokines release during exaggerated inflammatory response related to disease severity^23,24^. This conclusion is supported by the elevated PRISM-III scores in patients with high cortisol levels and the significant correlation between cortisol levels and PRISM-III scores reported by the current study.

In our study, the prevalence of CIRCI was 18.5% in comparison to 25.0% in another study on PICU patients with pediatric acute lung injury/acute respiratory distress syndrome^25^ and 39.6% and 44.4% in patients with severe sepsis and septic shock respectively^26^. In addition, our study found no association between CIRCI and the reported mortality rate in accordance with a previous study^27^.

Of note, patients with normal total serum cortisol levels had significantly higher mortality rate than those with CIRCI. A probable explanation for this is the significantly higher PRISM-III score in the former patients’ subgroup.

Conclusions of the present study are limited by the relatively small sample size. Considering the wide range of cortisol levels reported in the studied patients, it may be useful to add a control group of healthy volunteers.

## Conclusion

PRISM-III score and elevated basal total serum cortisol levels are significant predictors of mortality in the PICU. Although, CIRCI is prevalent in this population, it wasn’t associated with increased mortality rate.

## Data Availability

All data generated or analyzed during this study are included in this published article.
